# Equilibrium Analysis of a Yellow Fever Dynamical Model with Vaccination

**DOI:** 10.1155/2015/482091

**Published:** 2015-03-05

**Authors:** Silvia Martorano Raimundo, Marcos Amaku, Eduardo Massad

**Affiliations:** ^1^School of Medicine, University of São Paulo and LIM01 HC-FMUSP, Avenida Doutor Arnaldo 455, Cerqueira César, 01246-903 São Paulo, SP, Brazil; ^2^School of Veterinary Medicine and Zootechnology, University of São Paulo, Avenida Professor Doutor Orlando Marques de Paiva 87, Cidade Universitária, 05508 270 São Paulo, SP, Brazil; ^3^London School of Hygiene and Tropical Medicine, London University, Keppel Street, London WC1E 7HT, UK

## Abstract

We propose an equilibrium analysis of a dynamical model of yellow fever transmission in the presence of a vaccine. The model considers both human and vector populations. We found thresholds parameters that affect the development of the disease and the infectious status of the human population in the presence of a vaccine whose protection may wane over time. In particular, we derived a threshold vaccination rate, above which the disease would be eradicated from the human population. We show that if the mortality rate of the mosquitoes is greater than a given threshold, then the disease is naturally (without intervention) eradicated from the population. In contrast, if the mortality rate of the mosquitoes is less than that threshold, then the disease is eradicated from the populations only when the growing rate of humans is less than another threshold; otherwise, the disease is eradicated only if the reproduction number of the infection after vaccination is less than 1. When this reproduction number is greater than 1, the disease will be eradicated from the human population if the vaccination rate is greater than a given threshold; otherwise, the disease will establish itself among humans, reaching a stable endemic equilibrium. The analysis presented in this paper can be useful, both to the better understanding of the disease dynamics and also for the planning of vaccination strategies.

## 1. Introduction

Yellow fever (YF), a hemorrhagic fever caused by a* Flavivirus*, family* Flaviviridae *[1, 2], is characterized by fever, chills, loss of appetite, nausea, muscle pains particularly in the back, and headaches [3]. There are more than 200,000 infections and 30,000 deaths every year [3]. About 90% of YF cases occur in Africa [4], and a billion people live in an area of the world where the disease is common [3]. It also affects tropical areas of South America, but not Asia [3, 5, 6]. The number of cases of yellow fever has been increasing in the last 30 years [3, 7], probably due to fewer people being immune, more people living in cities, people moving frequently, and changing climate [3]. The origin of the disease is Africa, from where it spread in South America through the slave trade in the 17th century [8, 9].

The yellow fever virus was the first human virus discovered [10], and its family comprises approximately 70 viruses [2], most of which are transmitted by arthropod insects (hence the name arthropod borne viruses or arboviruses).

A safe and effective vaccine against yellow fever exists and some countries require vaccinations for travelers [3]. In rare cases (less than one in 200,000 to 300,000 doses), the vaccination can cause yellow fever vaccine-associated viscerotropic disease (YEL-AVD), which is fatal in 60% of cases, probably due to the genetic morphology of the immune system. Another possible side effect is an infection of the nervous system, which occurs in one in 200,000 to 300,000 cases, causing yellow fever vaccine-associated neurotropic disease (YEL-AND), which can lead to meningoencephalitis, fatal in less than 5% of cases [6]. In some rare circumstances, however, the fatality rate of vaccine induced diseases can reach alarming proportions, as observed recently by Mascheretti et al. [11], who found 1 death per million doses applied in a Southeastern Brazilian region.

In this paper, we propose an equilibrium analysis of a dynamical model of yellow fever transmission in the presence of a vaccine. Such a kind of analysis can be useful, both to the better understanding of the disease dynamics and also for the planning of vaccination strategies.

## 2. Model Formulation

The mathematical model described below addresses the transmission dynamics of an infectious agent in a homogeneous population in the presence of an imperfect vaccine. We consider a nonlinear system of ordinary differential equations involving the human and the vector—mosquitoes and their eggs—populations. The term “eggs” also includes the intermediate stages, such as larvae and pupae. It is also worth highlighting that the model proposed here is based on previous papers [12, 13], and we updated the model originally developed by Amaku et al., 2013 [14, 15], for the purpose of investigating the impact of vaccination on population. By including vaccination, in particular vaccines which may have serious adverse effects, the model may help the designing of realistic (from the cost and logistic point of view) vaccination strategies.

All variables and parameters in the human system will carry the subscript  *H*, while those in the vector system will carry one of the subscripts  *M*  (mosquitoes) or  *E*  (eggs). In our model the total human population, denoted by  *N*_*H*_, is split into four subclasses which are susceptible humans (*S*_*H*_), vaccinated humans (*V*_*H*_), infected humans (*I*_*H*_), and recovered (and immune) humans (*R*_*H*_), so that  *N*_*H*_ = *S*_*H*_ + *I*_*H*_ + *R*_*H*_ + *V*_*H*_. The total vector population, which is formed by both total mosquitoes population, denoted by  *N*_*M*_, and the total eggs population, denoted by  *N*_*E*_, are split into susceptible mosquitoes (*S*_*M*_), infected and latent mosquitoes (*L*_*M*_), infected and infectious mosquitoes (*I*_*M*_), and noninfected eggs (*S*_*E*_), so that  *N*_*M*_ = *S*_*M*_ + *L*_*M*_ + *I*_*M*_  and  *N*_*E*_ = *S*_*E*_.

**Figure 1:**
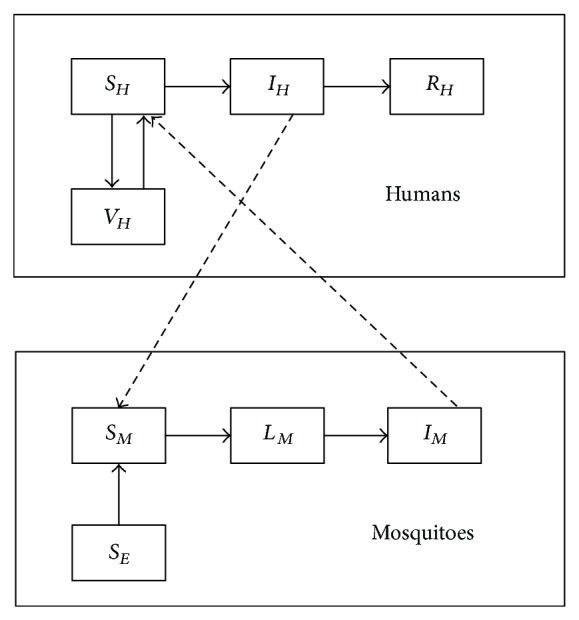
Schematic diagram of the yellow fever model (1).

A flow diagram of the model is depicted in Figure 1, and the associated variables and parameters are described in Tables 1 and 2, respectively (values from references [16–22]).

The model supposes a homogeneous mixing of human and mosquito population based on the idea that the mosquito has a human biting habit, so that each mosquito bite has an equal probability of transmitting the virus to the susceptible human in the population or acquiring infection from an infected human. The equations are derived based on the fact that, in presence of the yellow fever in the population, both mosquitoes and humans can infect each other upon contact. While an infected mosquito remains infected until death, it is assumed that infected humans can recover from the disease (see [23]). We define a logistic recruitment rate of humans, mosquitoes, and eggs, and all new born humans and newly emerged mosquitoes are susceptible (no vertical transmission; see [23]). Susceptible humans become infected through the bite by an infected mosquito and the susceptible mosquitoes become latent infected as result of biting infectious humans. Upon acquiring infection, the susceptible individuals move into the infected compartment. The incidence of new infections is given by the standard incidence (see [24, pp. 602]).

Deaths can occur amongst the human population, mosquitoes, and eggs, naturally. In contrast, in the presence of the yellow fever, the human population can either die due to the additional effects of the disease or recover. It is also assumed that recovered human individuals acquire immunity against reinfection, so that they do not acquire yellow fever for a second time.

Although there is a vaccine for yellow fever, it is expected that it is imperfect; that is, it does not offer 100% protection against infection in all population. Thus, it is instructive to assess the potential impact of an imperfect yellow fever vaccine.

After the duration of protection wanes down, the vaccinated individuals, *V*_*H*_, move to the susceptible class, *S*_*H*_, and they may then acquire a new infection. Hence, the vaccinated population is decreased by the waning of vaccine-induced immunity and by natural death.

Combining the above formulation and assumption, it follows that the model for the transmission dynamics of the yellow fever disease in the presence of an imperfect vaccine is given by the following system of nonlinear ordinary differential equations:

(1) dSHdt=−abIMSHNH−μHSH+rHNH1−NHκH−fHυHSH+ωHVH,dVHdt=fHυHSH−μH+ωHVH,dIHdt=abIMSHNH−μH+αH+γHIH,dRHdt=γHIH−μHRH,dNHdt=rHNH1−NHκH−μHNH−αHIH,dSMdt=pcSSE−μM+acIHNHSM,dLMdt=acSMIHNH−γM+μMLM,dIMdt=γMLM−μMIM,dNMdt=pcSSE−μMNM,dSEdt=rMNM1−SEκE−μE+pcSSE

with the conditions *N*_*H*_ = *S*_*H*_ + *V*_*H*_ + *I*_*H*_ + *R*_*H*_, *N*_*M*_ = *S*_*M*_ + *L*_*M*_ + *I*_*M*_, and *N*_*E*_ = *S*_*E*_ and the initial conditions *S*_*H*_(0) ≥ 0, *V*_*H*_(0) ≥ 0, *I*_*H*_(0) ≥ 0, *R*_*H*_(0) ≥ 0, *S*_*M*_(0) ≥ 0, *L*_*M*_(0) ≥ 0, *I*_*M*_(0) ≥ 0, *N*_*M*_(0) ≥ 0, and *S*_*E*_(0) ≥ 0. Note that the transmission from mosquitoes to humans is given by *ab*(*I*_*M*_/*N*_*H*_) (also called “force of infection”) and from humans to mosquitoes is given by *ac*(*I*_*H*_/*N*_*H*_). This means that the transmission from mosquito to humans depends on the number of infective mosquitoes, but the transmission from human to mosquitoes depends on the density of infective humans.

Since system (1) models human, mosquito, and eggs populations, it is assumed that all variables in the system are nonnegative. This assumption yields the epidemiologically feasible domain:

(2) Ω=SH,VH,IH,RH,SM,LM,IM,SE∈R8: SH≥0, VH≥0, IH≥0, RH≥0, 0≤SH+VH+IH+RH≤NH, SM≥0, LM≥0, IM≥0, 0≤SM+LM+IM≤NM, SE=NE≥0.

Since the right-hand sides of equations of system (1) and their partial derivatives are continuous in *Ω*, we will use the techniques described in [21] that there exist a unique solution *S*_*H*_(*t*) + *V*_*H*_(*t*) + *I*_*H*_(*t*) + *R*_*H*_(*t*) = *N*_*H*_(*t*), *S*_*M*_ + *L*_*M*_(*t*) + *I*_*M*_(*t*) = *N*_*M*_(*t*), and *S*_*E*_(*t*) = *N*_*E*_(*t*), for all *t* ≥ 0, satisfying the initial conditions specified within *Ω*, *S*_*H*_(0) + *V*_*H*_(0) + *I*_*H*_(0) + *R*_*H*_(0) = *N*_*H*_(0), *S*_*M*_(0) + *L*_*M*_(0) + *I*_*M*_(0) = *N*_*M*_(0), and *S*_*E*_(0) = *N*_*E*_(0), at time *t* = 0. It can also be verified that the given initial conditions make sure that *N*_*H*_ ≥ 0. Thus, the total population *N*_*H*_ remains positive and bounded for all finite time *t* > 0. Similar arguments can be applied to both mosquito and eggs equations with corresponding expressions. Therefore, all solutions of the model with initial conditions in *Ω* remain in *Ω* for all *t* ≥ 0, the region *Ω* is positively invariant with respect to model (1), and its solutions are considered epidemiologically and mathematically well posed in *Ω*.

## 3. The Existence of Equilibria

Our next result concerns the existence of equilibrium points of system (1) that are biologically feasible. Thus, we will find the equilibrium points of system (1) in the region *Ω* by setting right hand side of all equations in it as equal to zero. First of all, we will seek the conditions for the existence of the equilibria of system (1) which are biologically feasible.

From the second and fourth equations of (1) with the right-hand side equal to zero, it can be seen that the equilibrium points must satisfy, respectively, the following relations:

(3) $$\begin{array}{c} V_{H}^{*}=\rho_{\text{vac}}N_{H}^{*}\left[1-\frac{\left(\mu_{H}+\gamma_{H}\right)}{\mu_{H}}\frac{I_{H}^{*}}{N_{H}^{*}}\right], \end{array}$$

(4) $$\begin{array}{c} R_{H}^{*}=\frac{\gamma_{H}}{\mu_{H}}I_{H}^{*}, \end{array}$$

where

(5) $$\begin{array}{c} 0<\rho_{\text{vac}}=\frac{f_{H}\nu_{H}}{f_{H}\nu_{H}+\mu_{H}+\omega_{H}}<1, \end{array}$$

(6) $$\begin{array}{c} V_{H}^{*}>0⟺\frac{\;I_{H}^{*}}{N_{H}^{*}}<\frac{\mu_{H}}{\mu_{H}+\gamma_{H}}<1⟹I_{H}^{*}<N_{H}^{*}. \end{array}$$

Therefore, *V*_H_^*^ > 0 is always satisfied.

Substituting (3) and (4) into the third equation of system (1), we obtain

(7) $$\begin{array}{c} I_{M}^{*}=\frac{\left(\mu_{H}+\alpha_{H}+\gamma_{H}\right)I_{H}^{*}}{ab\left(1-\rho_{\text{vac}}\right)\left\{1-\left[\left(1+\gamma_{H}/\mu_{H}\right)\left(I_{H}^{*}/N_{H}^{*}\right)\right]\right\}}, \end{array}$$

(8) $$\begin{array}{c} I_{M}^{*}>0⟺\frac{\;I_{H}^{*}}{N_{H}^{*}}<\frac{\mu_{H\;}}{\mu_{H}+\gamma_{H}}<1⟹I_{H}^{*}<N_{H}^{*}. \end{array}$$

Hence, *I*_M_^*^ > 0 is also always satisfied.

From eighth and ninth equations of system (1), we obtain

(9) $$\begin{array}{c} L_{M}^{*}=\frac{\mu_{M}}{\gamma_{M}}I_{M}^{*}, \end{array}$$

(10) $$\begin{array}{c} N_{M}^{*}=\frac{pc_{S}}{\mu_{M}}S_{E}^{*}. \end{array}$$

From tenth equation of system (1), we get either *S*_E_^*^ = 0 or

(11) $$\begin{array}{c} S_{E}^{*}=k_{E}\left[1-\frac{\mu_{M}}{\mu_{M}^{\text{thres}}}\right], \end{array}$$

with *μ*_*M*_^thres^ = *r*_*M*_ *pc*_*S*_/(*μ*_*E*_ + *pc*_*S*_). From expression (11) it follows that

(12) $$\begin{array}{c} S_{E}^{*}>0⟺\mu_{M}<\mu_{M}^{\text{thres}}. \end{array}$$

Later we will see that for *S*_E_^*^ = 0 only the trivial equilibrium can exist, while for *S*_E_^*^ > 0, given by (11), both the trivial and the nontrivial equilibrium may exist for system (1).

Substituting (9) into the seventh equation of system (1), we obtain

(13) $$\begin{array}{c} I_{M}^{*}=\frac{acN_{M}^{*}\left(I_{H}^{*}/N_{H}^{*}\right)\;}{\left(ac\left(I_{H}^{*}/N_{H}^{*}\right)+\mu_{M}\right)\left(1+\mu_{M}/\gamma_{M}\right)}. \end{array}$$

From (7) and (13) we obtain either *I*_H_^*^ = 0 or

(14) IH∗=a2bcγM1−ρvacNM∗ −μH+αH+γHγM+μMμMNH∗ ·a2bcγM1+γHμHγM+μM1−ρvacNM∗NH∗   NM∗NH∗+acμH+αH+γHγM+μM−1.

From expression (14), it should be noted that, for *μ*_*M*_ < *μ*_*M*_^thres^, *I*_H_^*^ > 0 whenever

(15) $$\begin{array}{c} \frac{a^{2}bc\gamma_{M}N_{M}^{*}}{\mu_{M}\left(\mu_{H}+\alpha_{H}+\gamma_{H}\right)\left(\gamma_{M}+\mu_{M}\right)N_{H}^{*}}>\frac{1}{\left(1-\rho_{\text{vac}}\right)}, \end{array}$$

or

(16) 0<NH∗<a2bcpcSγMkEμM2μH+αH+γHγM+μMiiiiiii·1−μMμMthres1−ρvac=N1.

On the other hand, from fifth equation of system (1), we also get

(17) $$\begin{array}{c} I_{H}^{*}=\left[\frac{r_{H}}{\alpha_{H}}\left(1-\frac{N_{H}^{*}}{k_{H}}\right)-\frac{\mu_{H}}{\alpha_{H}}\right]N_{H}^{*}, \end{array}$$

and *I*_H_^*^ > 0 whenever

(18) $$\begin{array}{c} 0<N_{H}^{*}<\frac{k_{H}\left(r_{H}-\mu_{H}\right)}{r_{H}}=N_{2}\;\text{with}\;\;r_{H}>\mu_{H}. \end{array}$$

From expressions (16) and (18), one can note, however, that, *N*_2_ is the maximum value of *N*_H_^*^, so it follows that *N*_1_ < *N*_2_ and *I*_H_^*^ > 0 if and only if condition (18) holds. Furthermore, for *I*_H_^*^ > 0 the system (1) reaches an endemic equilibrium point.

In contrast, from (4), (9), (13), and (18), *I*_H_^*^ = 0 leads to

(19) $$\begin{array}{c} N_{H}^{*}=N_{2},\;\;I_{M}^{*}=0,\;\;L_{M}^{*}=0,\\ V_{H}^{*}=\rho_{\text{vac}}N_{H}^{*},\;\;S_{H}^{*}=N_{H}^{*}-V_{H}^{*},\;\;R_{H}^{*}=0, \end{array}$$

where *S*_E_^*^ = *N*_E_^*^ ≠ 0 and *S*_M_^*^ = *N*_M_^*^ ≠ 0 are also given by expressions (10) and (11), respectively. Therefore, for *I*_H_^*^ = 0 only the trivial equilibrium exists for system (1). To be more specific, for *S*_E_^*^ = *N*_E_^*^ ≠ 0 and *S*_M_^*^ = *N*_M_^*^ ≠ 0 the trivial equilibrium is given by the densities of humans and vectors, while for *S*_E_^*^ = *N*_E_^*^ = 0 and *S*_M_^*^ = *N*_M_^*^ = 0 the trivial equilibrium is given only by the density of humans.

Now, by substituting (14) in (18), it can be shown that the nontrivial equilibria of the model satisfy the following quadratic equation (in terms of *N*_H_^*^):

(20) $$\begin{array}{c} P\left(N_{H}^{*}\right)=Q_{2}{\left(N_{H}^{*}\right)}^{2}+Q_{1}N_{H}^{*}+Q_{0}=0, \end{array}$$

where

(21) $$\begin{array}{c} Q_{2}=ac\theta r_{H},\\ Q_{1}=\theta \left(1-\rho_{\text{vac}}\right)\left(1+\frac{\gamma_{H}}{\mu_{H}}\right)k_{H}\left(r_{H}-\mu_{H}\right)\mu_{M}\left(R_{0}-\Psi \right),\\ Q_{0}=\tau k_{H}\left(\mu_{H}+\gamma_{H}\right)\left(r_{H}^{\text{thres}}-r_{H}\right), \end{array}$$

with
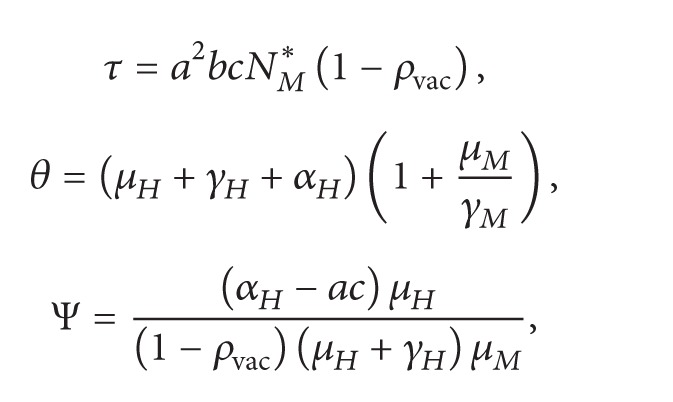
(22)

(23) $$\begin{array}{c} r_{H}^{\text{thres}}=\frac{\mu_{H}\left(\mu_{H}+\alpha_{H}+\gamma_{H}\right)\;}{\left(\mu_{H}+\gamma_{H}\right)}, \end{array}$$

(24) $$\begin{array}{c} R_{0}=\frac{a^{2}bcpc_{S}k_{E}r_{H}}{{\left(\mu_{M}\right)}^{2}\theta k_{H}\left(r_{H}-\mu_{H}\right)}\left(1-\frac{\mu_{M}}{\mu_{M}^{\text{thres}}}\right), \end{array}$$

with *μ*_*M*_^thres^ satisfying the condition given by (12).

The positive endemic equilibrium of model (1) is obtained by solving for *N*_H_^*^ from the quadratic (20) and substituting the results (positive values of *N*_H_^*^) into the expressions that give the coordinates of equilibrium point.

First, it is straightforward to note that *r*_*H*_^thres^ given by (23) and *θ* given by (22) are positive. Moreover, under our assumption Ψ < 0 and *Q*_1_ is also positive. Clearly, the coefficient *Q*_2_ of the quadratic equation (20) is always positive.

Also, note that the coefficient *Q*_0_ is positive either if *τ* > 0 and *r*_*H*_ < *r*_*H*_^thres^ or *τ* < 0 and *r*_*H*_ > *r*_*H*_^thres^. If *τ* < 0 then *μ*_*M*_ > *μ*_*M*_^thres^ and the positive endemic equilibrium does not exist (see (12)) for system (1). Later we will see that, for *μ*_*M*_ > *μ*_*M*_^thres^, the only equilibrium biologically feasible and stable is the equilibrium given by *P*_0_^*H*^.

Therefore, *Q*_0_ > 0 if and only if *τ* > 0 (i.e., *μ*_*M*_ < *μ*_*M*_^thres^) and *r*_*H*_ < *r*_*H*_^thres^. In this case, if *Q*_1_ > 0, the quadratic equation (20) does not have a positive solution. It follows then that system (1) does not have positive endemic equilibria whenever *r*_*H*_ < *r*_*H*_^thres^. In this case, note that the only equilibrium biologically feasible and stable for system (1) is the trivial equilibrium given by *P*_0_^*HM*^ (see (19)).

Otherwise, *τ* > 0 (i.e., *μ*_*M*_ < *μ*_*M*_^thres^) but *r*_*H*_ > *r*_*H*_^thres^; then *Q*_0_ < 0, so there is a unique positive real solution of the quadratic equation (20), for any value of *Q*_1_. Hence, for *r*_*H*_ > *μ*_*H*_ and *μ*_*M*_ < *μ*_*M*_^thres^, the system (1) has a unique positive endemic equilibrium when *r*_*H*_ > *r*_*H*_^thres^.

If *Q*_0_ = 0 (i.e., *r*_*H*_ = *r*_*H*_^thres^) and *Q*_1_ < 0 or *Q*_1_^2^ − 4*Q*_1_ *Q*_0_ = 0, then there is a unique positive real solution for the quadratic equation (20), so the system (1) has a unique positive endemic equilibrium when *r*_*H*_ = *r*_*H*_^thres^.

Therefore, for *r*_*H*_ > *μ*_*H*_, the above analysis indicates the possibility of the existence of the following positive equilibrium points for system (1):

- a disease-free equilibrium (DFE) defined only by human population, *P*_0_^*H*^ = (*S*_*H*_^0^, *V*_*H*_^0^, 0,0, *N*_*H*_^0^, 0,0, 0, 0, 0);
- a disease-free equilibrium (DFE) defined by both human and vector populations defined by *P*_0_^*HM*^ = (*S*_*H*_^0^, *V*_*H*_^0^, 0,0, *N*_*H*_^0^, *S*_*M*_^0^, 0,0, *N*_*M*_^0^, *S*_*E*_^0^);
- an endemic equilibrium (EE) point biologically feasible given by *P*_1_^*HM*^ = (*S*_H_^*^, *I*_H_^*^, *R*_H_^*^, *V*_H_^*^, *N*_H_^*^, *S*_M_^*^, *L*_M_^*^, *I*_M_^*^, *N*_M_^*^, *S*_E_^*^).

Quite apart from this, the existence of the positive equilibrium points for the system (1) can be also summarized as follows.

**Theorem 1 .**

Assuming that *r*_*H*_ > *μ*_*H*_ holds, model (1) has a unique disease-free equilibrium *P*_0_^*H*^ whenever *μ*_*M*_ > *μ*_*M*_^*thres*^. Otherwise, if *μ*_*M*_ < *μ*_*M*_^*thres*^ then model (1) has the trivial equilibrium *P*_0_^*HM*^ and the endemic equilibrium *P*_1_^*HM*^. For *r*_*H*_ < *r*_*H*_^*thres*^, the unique equilibrium that exists is given by the trivial equilibrium *P*_0_^*HM*^. Otherwise, for *r*_*H*_ > *r*_*H*_^*thres*^, both the trivial equilibrium, *P*_0_^*HM*^, and the endemic equilibrium, *P*_1_^*HM*^, exist.

Having found the scenarios in which there exist the equilibria for the system (1), it is instructive to analyse whether or not these equilibria are stable under any of these scenarios. Furthermore, together with the threshold vaccination rate, *ν*_*H*_^*C*^, and the reproduction number, *R*_vac_, we will see that each scenario can be used as a check for the existence, the uniqueness, and the stability of all equilibria. This is explored below for  *r*_*H*_ > *μ*_*H*_.

### 3.1. Disease-Free Equilibria

In the absence of the disease, that is, *R*_*H*_^0^ = *I*_*H*_^0^ = *I*_*M*_^0^ = *L*_*M*_^0^ = 0 and for *r*_*H*_ > *μ*_*H*_, model (1) has two disease-free equilibria given by *P*_0_^*H*^ and *P*_0_^*HM*^. Thus, if *μ*_*M*_ < *μ*_*M*_^thres^, then the disease-free equilibrium is given by *P*_0_^*HM*^:

(25) $$\begin{array}{c} S_{H}^{0}=\frac{k_{H}\left(r_{H}-\mu_{H}\right)}{r_{H}}\left(1-\rho_{\text{vac}}\right),\\ V_{H}^{0}=\rho_{\text{vac}}N_{H}^{0},\\ N_{H}^{0}=\frac{k_{H}\left(r_{H}-\mu_{H}\right)}{r_{H}},\\ S_{M}^{0}=N_{M}^{0}=\frac{pc_{S\;\;}}{\mu_{M}}S_{E}^{0},\\ S_{E}^{0}=k_{E}\left[1-\frac{\;\mu_{M}}{\mu_{M}^{\text{thres}}}\right], \end{array}$$

where  *ρ*_vac_  is defined by expression (5). In contrast, if *μ*_*M*_ > *μ*_*M*_^thres^, then the only equilibrium biologically viable is *P*_0_^*H*^, which is also given by (25), but with *S*_*M*_^(0)^ = *N*_*M*_^(0)^ = *S*_*E*_^(0)^ = *N*_*E*_^(0)^ = 0.

To establish the stability of both trivial equilibrium, the Jacobian of the system (1) is computed and evaluated at both *P*_0_^*H*^ and *P*_0_^*HM*^. We will discuss the properties of both trivial equilibrium points making an elementary row-transformation for the Jacobian matrix.

Evaluating the system's Jacobian at *P*_0_^*H*^, the local stability of *P*_0_^*H*^ is straightforward determined by the six eigenvalues given by *τ*_1_ = *τ*_2_ = −*μ*_*M*_, *τ*_3_ = −*μ*_*H*_, *τ*_4_ = −(*μ*_*M*_ + *γ*_*M*_), *τ*_5_ = −(*μ*_*H*_ + *α*_*H*_ + *γ*_*H*_), and *τ*_6_ = (*μ*_*H*_ − *r*_*H*_) < 0 since *r*_*H*_ > *μ*_*H*_. The other eigenvalues are expressed as the roots of the following submatrix:

(26) MP0H=−fHνH+μHωH00fHνH−ωH+μH0000−  μMpcS00rM−μE+pcS,

where

(27) $$\begin{array}{c} M_{H}^{0}=\left[\begin{array}{cc} -\left(f_{H}\nu_{H}+\mu_{H}\right) & \omega_{H}\\ f_{H}\nu_{H} & -\left(\omega_{H}+\mu_{H}\right) \end{array}\right],\\ M_{H}^{1}=\left[\begin{array}{cc} -\mu_{M} & pc_{S}\\ r_{M} & -\left(\mu_{E}+pc_{S}\right) \end{array}\right]. \end{array}$$

It is easy to verify that both the traces of the matrices tr⁡(*M*_*H*_^0^) and tr⁡(*M*_*H*_^1^) are always negative. Moreover, the determinant of the matrix, det⁡(*M*_*H*_^0^), is always positive, but det⁡(*M*_*H*_^1^) is positive if and only if *μ*_*M*_ > *μ*_*M*_^thres^. In other words, it means that the four eigenvalues of matrix *M* ^*P*_0_^*H*^^ are either negative or have negative real part whenever *μ*_*M*_ > *μ*_*M*_^thres^.

Therefore, all the eigenvalues of the characteristic equation associated with the system (1) at *P*_0_^*H*^ have negative real parts if and only if *μ*_*M*_ > *μ*_*M*_^thres^ and *r*_*H*_ > *μ*_*H*_. We state then the following result.

**Lemma 2 .**

For *r*_*H*_ > *μ*_*H*_, the disease-free equilibrium *P*_0_^*H*^ of model (1) is globally asymptotically stable if *μ*_*M*_ > *μ*_*M*_^*thres*^. Otherwise, *P*_0_^*H*^ is unstable.

The stability of the disease-free equilibrium *P*_0_^*HM*^ is now examined by linearizing the system (1) around *P*_0_^*HM*^. The characteristic equation of the Jacobian matrix of the system (1) at *P*_0_^*HM*^ is given by

(28) Λ1−μM−λ−μH+αH+γH−λ−μM+γM−λ  +χ1MP0HM=0,

where

(29) $$\begin{array}{c} \Lambda_{1}=\left[-\mu_{H}-\lambda \right]\left[-\mu_{M}-\lambda \right]\left[\left(\mu_{H}-r_{H}\right)-\lambda \right], \end{array}$$

(30) $$\begin{array}{c} \chi_{1}=a^{2}bc\gamma_{M}\frac{S_{H}^{0}S_{M}^{0}}{{\left(N_{H}^{0}\right)}^{2}}, \end{array}$$

with *S*_*H*_^0^,  *S*_*M*_^0^, and *N*_*H*_^0^ given by (25) and

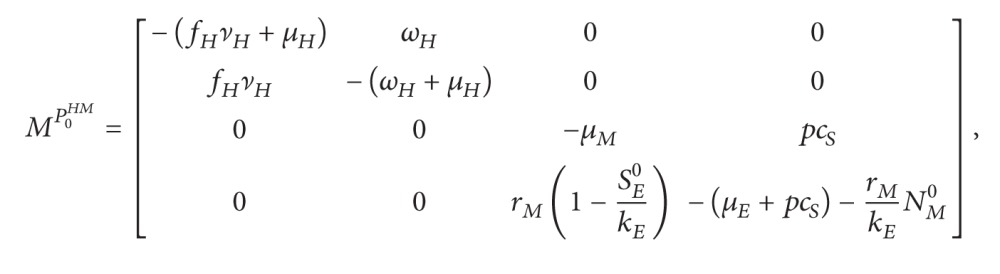
(31)where *M*_*HM*_^0^ = *M*_*H*_^0^ (see (27)) and

(32) $$\begin{array}{c} M_{HM}^{1}=\left[\begin{array}{cc} -\mu_{M} & pc_{S}\\ r_{M}\left(1-\frac{S_{E}^{0}}{k_{E}}\right) & -\left(\mu_{E}-pc_{S}\right)-\frac{r_{M}}{k_{E}}N_{M}^{0} \end{array}\right]. \end{array}$$

From (29) it is easy to verify that *λ*_1_ = −*μ*_*H*_, *λ*_2_ = −*μ*_*M*_, *λ*_3  _ = (*μ*_*H*_ − *r*_*H*_) < 0, since *r*_*H*_ > *μ*_*H*_. Moreover, from (27) and (32), both the traces of the matrices tr⁡(*M*_*HM*_^0^) and tr⁡(*M*_*HM*_^1^) are always negative; the determinant of the matrix, det⁡(*M*_*HM*_^0^), is also always positive, but det⁡(*M*_*HM*_^1^) is positive if and only if *μ*_*M*_ < *μ*_*M*_^thres^. Therefore, the four eigenvalues of matrix *M* ^*P*_0_^*HM*^^ are either negative or have negative real parts if and only if *μ*_*M*_ < *μ*_*M*_^thres^.

The other three eigenvalues are associated with the third degree equation in (28) given by

(33) −μM−λ−μH+αH+γH−λ−μM+γM−λ  +χ1=0.

It can be seen after some calculations that the polynomial (33) is equivalent to

(34) $$\begin{array}{c} \lambda^{3}+g_{2}\lambda^{2}+g_{1}\lambda +g_{0}=0, \end{array}$$

where

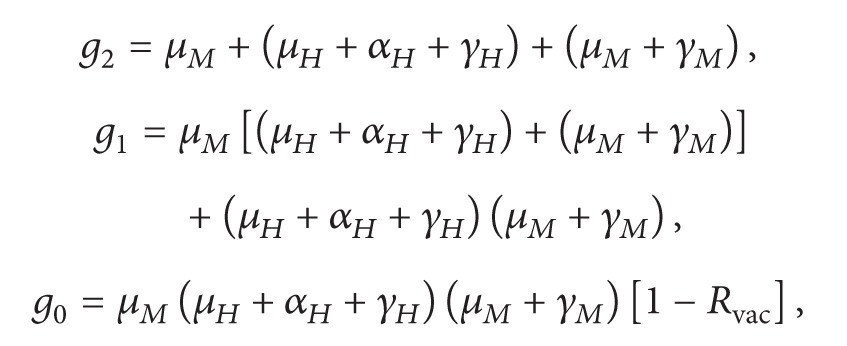
(35)

(36) $$\begin{array}{c} R_{\text{vac}}=R_{0}\left[1-\rho_{\text{vac}}\right], \end{array}$$

with *ρ*_vac_ < 1 given by (5); *R*_0_ is the threshold quantity or the basic reproductive number of the diseases defined by (23) and *R*_vac_ < *R*_0_. It is worth remembering that *P*_0_^*HM*^ given by (25) exists whenever *r*_*H*_ > *μ*_*H*_ and *μ*_*M*_ < *μ*_*M*_^thres^, so *R*_0_ > 0 and *R*_vac_ > 0.

By using the Routh-Hurwitz criteria for the polynomial (33), it follows that *g*_2_ > 0, *g*_1_ > 0, and *g*_0_ > 0 if and only if *R*_vac_ < 1 and *g*_1_ *g*_2_ − *g*_0_ > 0. Therefore, the polynomial (33) has negative (or has negative real part) roots if *R*_vac_ < 1.

Hence, for *r*_*H*_ > *μ*_*H*_, all the eigenvalues of the characteristic equation associated with the system (1) at *P*_0_^*HM*^ are negative or have negative real parts if and only if *μ*_*M*_ < *μ*_*M*_^thres^ and *R*_vac_ < 1. Hence, the disease-free equilibrium *P*_0_^*HM*^ is locally asymptotically stable when *R*_vac_ < 1 and *μ*_*M*_ < *μ*_*M*_^thres^.

It is worth remembering that if *μ*_*M*_ < *μ*_*M*_^thres^ and *r*_*H*_ < *r*_*H*_^thres^ there are no positive solutions of the quadratic equation (20) and thus there is no endemic equilibrium of system (1). However, by (19), it follows that the disease-free equilibrium *P*_0_^*HM*^ is the only equilibrium point that exists when *r*_*H*_ < *r*_*H*_^thres^. Otherwise, for *r*_*H*_ > *r*_*H*_^thres^, there is a unique positive real solution of the quadratic equation (20) which indicates the possibility of a unique positive endemic equilibrium given by *P*_1_^*HM*^. On the other hand, from (25) it can be also noted that the existence of the disease-free equilibrium *P*_0_^*HM*^ does not depend on *r*_*H*_^thres^, and thus *P*_0_^*HM*^ could also exist in this case. Therefore, the disease-free equilibrium *P*_0_^*HM*^ coexists with an endemic equilibrium *P*_1_^*HM*^ whenever *r*_*H*_ > *r*_*H*_^thres^.

It follows from above analyses that the disease-free equilibrium *P*_0_^*HM*^ is a unique equilibrium which is locally asymptotically stable whenever *μ*_*M*_ < *μ*_*M*_^thres^ and *r*_*H*_ < *r*_*H*_^thres^. In such a scenario, disease elimination would depend upon the birth rate of humans, *r*_*H*_, and the mosquitoes natural mortality rate  *μ*_*M*_. Otherwise, if *μ*_*M*_ < *μ*_*M*_^thres^ and *r*_*H*_ > *r*_*H*_^thres^, then *P*_0_^*HM*^ is locally asymptotically stable when *R*_vac_ < 1. The epidemiological implication of this is that the requirements of *μ*_*M*_ < *μ*_*M*_^thres^ and *r*_*H*_ > *r*_*H*_^thres^ are, although necessary, no longer sufficient for disease elimination. The disease elimination would depend upon the vaccination coverage (*υ*_*H*_) and thus will eliminate the disease from the population if the usage of an imperfect vaccine results in making (and keeping) *R*_vac_ < 1.

For the case when *μ*_*M*_ < *μ*_*M*_^thres^, for *r*_*H*_ < *r*_*H*_^thres^ the quadratic equation has no positive solution. Hence, system (1) has no positive solution (endemic equilibrium) for *r*_*H*_ < *r*_*H*_^thres^. As the only existing equilibrium for *μ*_*M*_ < *μ*_*M*_^thres^ is the trivial one, *P*_0_^*HM*^, then we can conjecture that, for *r*_*H*_ < *r*_*H*_^thres^, *P*_0_^*HM*^ is the only possible equilibrium for system (1), being, therefore, stable. On the other hand, for *r*_*H*_ > *r*_*H*_^thres^ the quadratic equation has a positive solution and hence, system (1) shows an endemic equilibrium, *P*_1_^*HM*^. As the existence of *P*_0_^*HM*^, however, does not depend on *r*_*H*_^thres^ (see (25)), we can state that *P*_0_^*HM*^ also exists for *r*_*H*_ > *r*_*H*_^thres^. In addition, if *R*_vac_ < 1, then *P*_0_^*HM*^ is stable. Note that, in all cases, the mosquito's mortality rate *μ*_*M*_ is the critical parameter, in the absence of vaccination, determining the stability of the disease.

How about the case when *R*_vac_ > 1, *P*_0_^*HM*^ becomes unstable? In this case, the critical value of vaccination is given by (38); that is, if *υ*_*H*_ > *υ*_*H*_^thres^, then *P*_0_^*HM*^ is stable. In contrast, if *υ*_*H*_ < *υ*_*H*_^thres^, then *P*_0_^*HM*^ is unstable and *P*_1_^*HM*^ becomes stable.

We establish then the following stability result for the system (1).

**Lemma 3 .**

For *r*_*H*_ > *μ*_*H*_ and *μ*_*M*_ < *μ*_*M*_^*thres*^ the disease-free equilibrium of the model (1), *P*_0_^*HM*^, exists and it is locally asymptotically stable if

- *R*_*vac*_ < 1 and *r*_*H*_ > *r*_*H*_^*thres*^,
- *r*_*H*_ < *r*_*H*_^*thres*^,
- otherwise, *P*_0_^*HM*^ is unstable.

The quantity *R*_vac_ given by (36) is called the vaccinated reproduction number, since it represents the expected average number of new infections produced by a single infective when introduced into a human community where a fraction of the susceptible population has been vaccinated. For a disease in which the susceptible population is vaccinated it has been demonstrated that *R*_vac_, which is the basic reproduction number *R*_0_ [26] modified by vaccination, must be reduced below one in order to ensure that the disease dies out [27]. If there is no vaccination, then *R*_vac_ = *R*_0_. Therefore, the aim of the vaccination must be to reduce *R*_vac_ below one and to provide prolonged protection against the infection.

Now, expression (36) for *R*_vac_ can be written in terms of *S*_*M*_^0^, *N*_*H*_^0^, and *S*_*H*_^0^ such as

(37) $$\begin{array}{c} R_{\text{vac}}=\frac{a^{2}bc\gamma_{M}\left(S_{M}^{0}/N_{H}^{0}\right)}{\left(\mu_{H}+\alpha_{H}+\gamma_{H}\right)\left(\mu_{M}+\gamma_{M}\right)\left(\mu_{M}\right)}\frac{S_{H}^{0}}{N_{H}^{0}}. \end{array}$$

Setting *R*_vac_ = 1, and solving (37) for *υ*_*H*_, the threshold vaccination rate is found to be

(38) $$\begin{array}{c} \upsilon_{H}^{\text{thres}}=\frac{\left(\mu_{H}+\omega_{H}\right)}{f_{H}}\left(R_{0}-1\right). \end{array}$$

It is worth remembering that *R*_0_ > 0 whenever *r*_*H*_ > *μ*_*H*_ and *μ*_*M*_ < *μ*_*M*_^thres^ (see (24)). More than that, *R*_0_ < 1 implies *R*_vac_ < 1 (see (36)), according to Lemma 3. *P*_0_^*HM*^ could be stable. Therefore, the disease eradication can be attainable independently of vaccination coverage, that is, even when *υ*_*H*_^thres^ = 0.

In contrast, if *R*_  0_ > 1, *υ*_*H*_^thres^ is positive but we also have either *R*_vac_ > 1 or *R*_vac_ < 1. In this situation, the disease could be eliminated whenever *υ*_*H*_ > *υ*_*H*_^thres^ and *P*_0_^*HM*^ is globally asymptotically stable. Otherwise, when *υ*_*H*_ < *υ*_*H*_^thres^, the use of an imperfect vaccine will fail to eliminate the disease from the community, *P*_0_^*HM*^ is unstable, the disease will persist in the community, and *P*_1_^*HM*^ becomes globally asymptotically stable.

Finally, concerning the analysis of the polynomial (20), Lemmas 2 and 3, we can establish the following conjecture for the system (1).

**Conjecture 4 .**

For *r*_*H*_ > *μ*_*H*_ the disease-free equilibrium *P*_0_^*H*^ is globally asymptotically stable if *μ*_*M*_ > *μ*_*M*_^*thres*^. If *μ*_*M*_ < *μ*_*M*_^*thres*^, *P*_0_^*H*^ becomes unstable and for *r*_*H*_ < *r*_*H*_^*thres*^, *P*_0_^*HM*^ is globally asymptotically stable equilibrium point. If *r*_*H*_ > *r*_*H*_^*thres*^, then *P*_0_^*HM*^ is globally asymptotically stable if *υ*_*H*_ > *υ*_*H*_^*thres*^. Otherwise, if *υ*_*H*_ < *υ*_*H*_^*thres*^, then *P*_0_^*HM*^ becomes unstable and the globally asymptotically equilibrium point stable is then given by *P*_1_^*HM*^.

The stability of the equilibrium points can then be summarized in Table 3.

As it will be explained in detail below, it is important to point out that by considering our model (1) without vaccine, that is, taking *ν*_*H*_ = *f*_*H*_ = *ω*_*H*_ = 0, we have *ρ*_vac_ = 0; the first term of expression (15) defines then the basic reproduction number used to gauge the severity of an epidemic; it will be denoted by *R*_0_. Therefore, according to inequality (15), *I*_H_^*^ > 0 whenever

(39) $$\begin{array}{c} \rho_{\text{vac}}<1-\frac{1}{R_{0}\;}. \end{array}$$

If *R*_0_ < 1, *ρ*_vac_ < 0, from (36), *R*_vac_ > 1. If *R*_0_ = 1, *ρ*_vac_ = 0, from (36), *R*_vac_ = *R*_0_. If *R*_0_ > 1, 0 < *ρ*_vac_ < 1, from (36), either *R*_vac_ > 1 or *R*_0_ < 1.

From (36) if vaccine has no effect, *ω*_*H*_ → *∞* (immune protection duration, *D* = 1/*ω*_*H*_ → 0), then *R*_vac_ increases (*r*_*H*_ < *r*_*H*_^thres^  or *r*_*H*_ > *r*_*H*_^thres^). If the vaccine induces lifelong immunity, then *ω*_*H*_ = 0 (*D* → *∞*), 0 < *ρ*_vac_ ≤ 1, *R*_vac_ > 1, or *R*_0_ < 1 (see (36)).

## 4. Numerical Analysis

The numerical analysis of the stability of the equilibria was done with the parameters of the model fixed at the baseline values indicated in Table 2. We explore the implications of variable vaccination coverage (*υ*_*H*_), birth rate of humans (*r*_*H*_), and mosquitoes natural mortality rate (*μ*_*M*_) which are chosen for simulations purposes only, so we can illustrate our theoretical results. For the baseline parameters values in Table 2, we have *r*_*H*_^thres^ = 0.000035085, *μ*_*M*_^thres^ = 4.751131, and *υ*_*H*_^thres^ = 8.0117.

**Figure 2:**
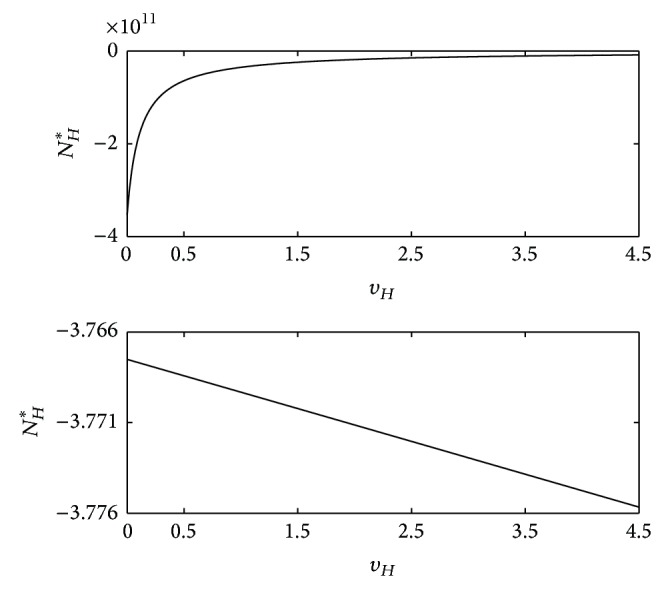
For  *μ*_*M*_ < *μ*_*M*_^thres^ and *r*_*H*_ < *r*_*H*_^thres^, the quadratic equation *P*(*N*_H_^*^) is plotted versus *υ*_*H*_, corresponding to two negative (which is not biologically relevant) real roots.

**Figure 3:**
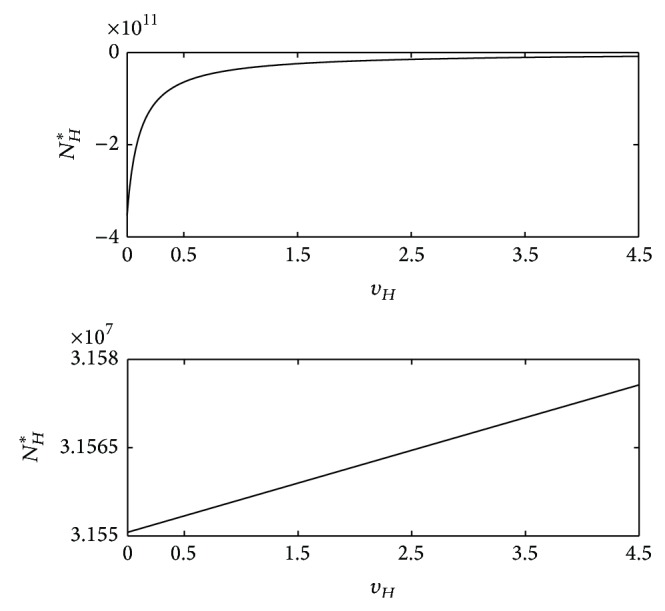
For *μ*_*M*_ < *μ*_*M*_^thres^ and *r*_*H*_ > *r*_*H*_^thres^, the quadratic equation *P*(*N*_H_^*^) is plotted versus *υ*_*H*_, corresponding to one negative (which is not biologically relevant) and one positive real roots.

Figures 2 and 3 show the graph of (20), with *P*(*N*_H_^*^) plotted versus *υ*_*H*_.  Figure 2 shows the graph for *μ*_*M*_ < *μ*_*M*_^thres^ and *r*_*H*_ < *r*_*H*_^thres^, with increasing values of *υ*_*H*_. One can see two real negative roots of (20), which are not biologically viable for the system (1). Therefore, the unique equilibrium point that is biologically viable and locally asymptotically stable is given by *P*_0_^*HM*^ (see (19)). In this case, the disease can be eradicated from the population.  Figure 3 shows the graph for *μ*_*M*_ < *μ*_*M*_^thres^ and *r*_*H*_ > *r*_*H*_^thres^, with increasing values of *υ*_*H*_. One can see one positive and one negative real root of (20). Hence, the system (1) has a unique endemic equilibrium *P*_1_^*HM*^, and the disease persists at an endemic level. Parameter values used are as given in Table 2 (baseline values), except for *υ*_*H*_.

**Figure 4:**
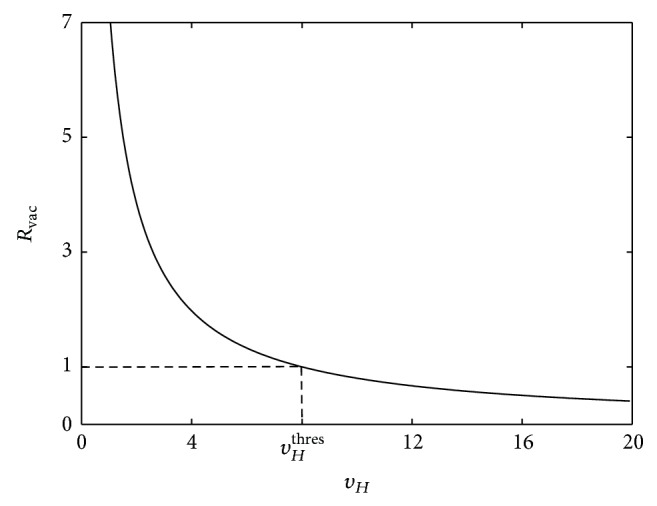
For *μ*_*M*_ < *μ*_*M*_^thres^ and *r*_*H*_ > *r*_*H*_^thres^. If *υ*_*H*_ > *υ*_*H*_^thres^, then *R*_vac_ < 1; if *υ*_*H*_ < *υ*_*H*_^thres^, then *R*_vac_ > 1.

Figure 4 shows the graph of (37) for *μ*_*M*_ < *μ*_*M*_^thres^ and *r*_*H*_ > *r*_*H*_^thres^, with increasing values of *υ*_*H*_. Parameter values used are as given in Table 2 (baseline values), except for *υ*_*H*_. For *υ*_*H*_ > *υ*_*H*_^thres^, *R*_vac_ < 1; the disease is then eradicated from population (*P*_0_^*HM*^ is locally asymptotically stable). For *υ*_*H*_ < *υ*_*H*_^thres^, then *R*_vac_ > 1; the disease persists into the population (*P*_1_^*HM*^ is the locally asymptotically stable).

**Figure 5:**
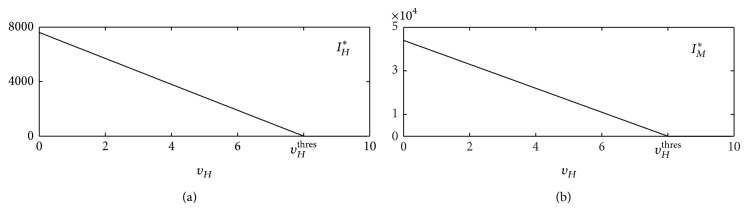
Prevalence of (a) infectious individuals (*I*_H_^*^) and (b) infectious mosquitoes (*I*_M_^*^) as a function of *υ*_*H*_. *P*_0_^*HM*^  is a unique equilibrium globally asymptotically stable for *υ*_*H*_ > *υ*_*H*_^thres^ and *P*_1_^*HM*^ is globally asymptotically stable for *υ*_*H*_ < *υ*_*H*_^thres^.

Figure 5 shows the prevalence of infectious individuals as a function of *υ*_*H*_. All parameters values used are given in Table 2 (baseline values), except for *υ*_*H*_. For *υ*_*H*_ > *υ*_*H*_^thres^, *I*_H_^*^ = 0, and *I*_M_^*^ = 0; thus, *P*_0_^*HM*^ is globally asymptotically stable. For *υ*_*H*_ < *υ*_*H*_^thres^, then *I*_H_^*^ ≠ 0 and *I*_M_^*^ ≠ 0 and *P*_1_^*HM*^ is globally asymptotically stable.

**Figure 6:**
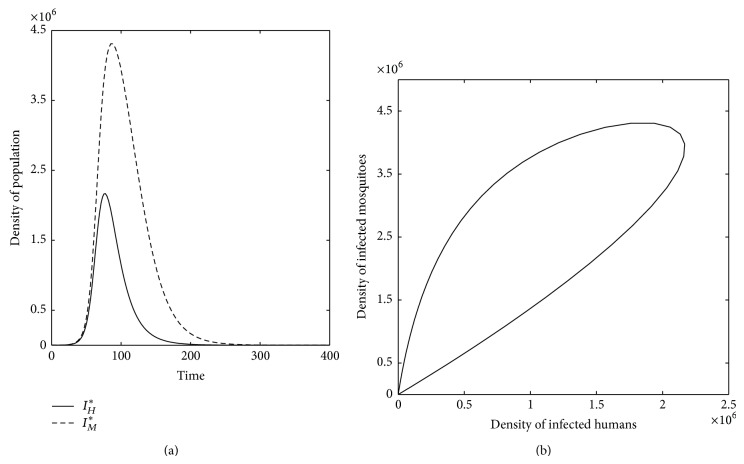
For *μ*_*M*_ < *μ*_*M*_^thres^ and *r*_*H*_ < *r*_*H*_^thres^, *P*_0_^*HM*^ is a unique equilibrium globally asymptotically stable for any vaccination coverage, *υ*_*H*_. (a) Profile of population of both infectious humans (*I*_H_^*^) and mosquitoes (*I*_M_^*^). (b) *I*_H_^*^ is plotted versus *I*_M_^*^. The system approaches *I*_H_^*^ = 0 and *I*_M_^*^ = 0, that is, the system approaches DFE, *P*_0_^*HM*^.

**Figure 7:**
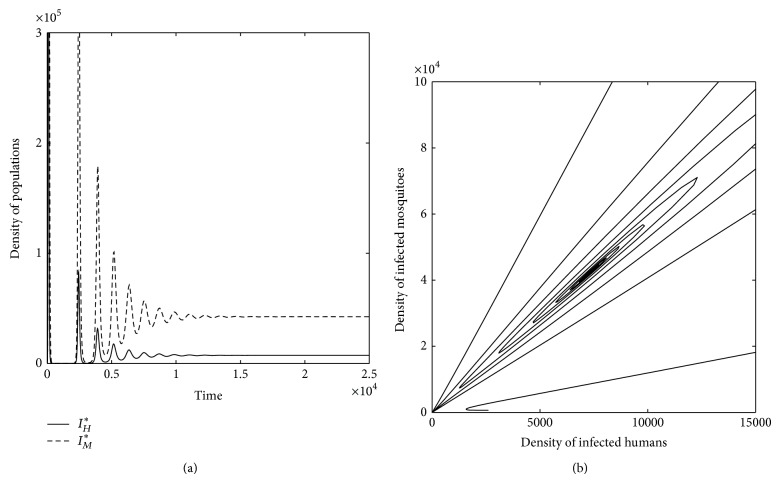
For *μ*_*M*_ < *μ*_*M*_^thres^ and *r*_*H*_ > *r*_*H*_^thres^, *P*_1_^*HM*^ is a unique equilibrium locally asymptotically stable for *υ*_*H*_ < *υ*_*H*_^thres^. (a) Profile of populations of both infectious humans (*I*_H_^*^) and mosquitoes (*I*_M_^*^). (b) *I*_H_^*^ is plotted versus *I*_M_^*^. The system approaches *I*_H_^*^ ≠ 0 and *I*_M_^*^ ≠ 0, that is, the system approaches endemic equilibrium, *P*_1_^*HM*^.

Figures 6 and 7 show the profiles of both infectious populations *I*_H_^*^ and *I*_M_^*^. In Figure 6, for *μ*_*M*_ < *μ*_*M*_^thres^ and *r*_*H*_ < *r*_*H*_^thres^ there is no positive real solution of *P*(*N*_H_^*^) and the DFE *P*_0_^*HM*^ is globally asymptotically stable. In Figure 7, for *μ*_*M*_ < *μ*_*M*_^thres^ and *r*_*H*_ > *r*_*H*_^thres^ the quadratic equation *P*(*N*_H_^*^) has one positive real solution. For *υ*_*H*_ < *υ*_*H*_^thres^, the endemic equilibrium *P*_1_^*HM*^ is, therefore, a unique equilibrium globally asymptotically stable. Parameter values used are as given in Table 2 (baseline values), except for *μ*_*M*_, *r*_*H*_, and *υ*_*H*_.

## 5. Sensitivity Analysis

In this section, we present the sensitivity analysis of the model to find out the degree to which the parameters influence the outputs of the model. Using the equation described in [15, 28, 29], we investigate only two of the most significant epidemiological concepts that affect the disease dynamics: the force of infection and the prevalence of infection.

The force of infection is defined as [28, 29]

(40) $$\begin{array}{c} \lambda =ab\frac{I_{M}^{*}}{N_{H}^{*}}, \end{array}$$

where *a* and *b* are given in Table 2. Thus, substituting (7) into expression (40) gives

(41) $$\begin{array}{c} \lambda =\frac{\left(\mu_{H}+\alpha_{H}+\gamma_{H}\right)\left(I_{H}^{*}/N_{H}^{*}\right)}{\left(1-\rho_{\text{vac}}\right)\left\{1-\left(1+\gamma_{H}/\mu_{H}\right)\left(I_{H}^{*}/N_{H}^{*}\right)\right\}}. \end{array}$$

After some simple algebraic manipulations, (14) reduces to

(42) $$\begin{array}{c} \frac{I_{H}^{*}}{N_{H}^{*}}=\frac{\left(1-\rho_{\text{vac}}\right)R^{*}-1}{k_{1}\left(1-\rho_{\text{vac}}\right)R^{*}+k_{2}}, \end{array}$$

where *k*_1_ = (1 + *γ*_*H*_/*μ*_*H*_)(*μ*_*M*_ + *γ*_*M*_), *k*_2_ = *ac*/*μ*_*M*_, and the basic reproduction number of the diseases, *R*_0_, is being approximated by

(43) $$\begin{array}{c} R^{*}=\frac{a^{2}bc\gamma_{M}\left(S_{M}^{*}/N_{H}^{*}\right)}{\left(\mu_{H}+\alpha_{H}+\gamma_{H}\right)\left(\mu_{M}+\gamma_{M}\right)\left(\mu_{M}\right)}, \end{array}$$

since *N*_*H*_^0^≅*N*_H_^*^ and *S*_*M*_^0^≅*S*_M_^*^ (see (37)).

In fact, *S*_M_^*^ and *N*_H_^*^ are smaller than *S*_*M*_^0^ and *N*_*H*_^0^, respectively. This is due to the fact that, in the endemic equilibrium, the disease “consumes” the susceptible and total population. As the prevalence of yellow fever is typically low, the approximation holds.

Note that since 0 < *ρ*_vac_ < 1, (42) is satisfied whenever *ρ*_vac_ < 1 − 1/*R* ^*^, that is, whenever *R* ^*^ > 1. In this case, the endemic equilibrium *P*_1_^*HM*^ is a unique equilibrium which is locally asymptotically stable. Moreover, we define *I*_H_^*^/*N*_H_^*^, given by (42), as a prevalence for model (1), and denote it by prev.

Now, applying the expression given by [15, 28, 29] to estimate the sensitivity of a variables *V*_*i*_ to the parameters *θ*_*j*_,

(44) $$\begin{array}{c} \frac{\Delta V_{i}}{V_{i}}=\frac{\theta_{j}}{V_{i}}\frac{\partial V_{i}}{\partial \theta_{j}}\frac{\Delta \theta_{j}}{\theta_{j}}, \end{array}$$

we can then calculate the sensitivity of both the force of infection *λ* (see (40)) and the prevalence (see (42)), for the parameter *ρ*_vac_, which are given by

(45) $$\begin{array}{c} \frac{\Delta \lambda }{\lambda }=\frac{\rho_{\text{vac}}}{\lambda }\frac{\left(\mu_{H}+\alpha_{H}+\gamma_{H}\right)}{\left[\left(1+\gamma_{H}/\mu_{H}\right)\left(\rho_{\text{vac}}+\text{prev}-1\right)-1\right]}\frac{\Delta \rho_{\text{vac}}}{\rho_{\text{vac}}},\\ \frac{\Delta \text{prev}}{\text{prev}}=\frac{\rho_{\text{vac}}}{\text{prev}}\frac{\Delta \rho_{\text{vac}}}{\rho_{\text{vac}}}, \end{array}$$

with Δ*ρ*_vac_/*ρ*_vac_ = 0.01.

Finally, applying (45) to estimate the sensitivity of force of infection and the prevalence to the vaccination effort (*ρ*_vac_) we can show that for every 1% of variation in *ρ*_vac_ results in a variation of approximated 0.6% and 844% in *λ* and prev, respectively. Therefore, the prevalence is 1400 more sensitive to vaccination than the force of infection. All parameters values used are given in Table 2 (baseline values).

## 6. Summary and Conclusions

In this paper, we have formulated a model for yellow fever disease with both human and vector populations as variables. We found four threshold parameters that control the development of the disease and the infectious status of the human population in the presence of a preventive vaccine whose protection may wane over time.

Our analysis is based on the assumption that the growth rate of the human population is positive, that is, *r*_*H*_ − *μ*_*H*_ > 0, which is the case of the yellow fever affected populations. We, therefore, can conclude the following:

(a) if the mortality rate of the mosquitoes is greater than the threshold, *μ*_*M*_ > *μ*_*M*_^thres^, then the disease is naturally (without intervention) eradicated from the population;
(b) if, in contrast, the mortality rate of the mosquitoes is less than the threshold, *μ*_*M*_ < *μ*_*M*_^thres^, then the disease is eradicated from the populations only when the growing rate of humans is less than a threshold, *r*_*H*_ < *r*_*H*_^thres^. Otherwise, *r*_*H*_ > *r*_*H*_^thres^; then the disease is eradicated only if *R*_vac_ < 1;
(c) in case *R*_vac_ > 1, then the disease will be eradicated from the human population if the vaccination rate is greater than a threshold, *υ*_*H*_ > *υ*_*H*_^thres^. Otherwise, *υ*_*H*_ < *υ*_*H*_^thres^; then the disease will establish itself among humans, reaching a stable endemic equilibrium. This conclusion derives from a rearrangement of (37); that is,
  (46) $$\begin{array}{c} R_{\text{vac}}=R_{0}\left[1-\rho_{\text{vac}}\right]>1⟹\left(1-\rho_{\text{vac}}\right)>\frac{1}{R_{0}}=\frac{S^{*}}{N}; \end{array}$$
 or in words, the vaccination effort should be such that 1 minus it should be greater than the proportion of susceptible at equilibrium;
(d) the prevalence is 1400 more sensitive to vaccination than the force of infection.

The model and analyses presented in this paper are intended to serve as a framework for testing alternative vaccination schedules taking into account the vaccine and disease induced mortality rates. This will be the subject of a future publication

## Figures and Tables

**Table 1:** Description of the variables of the yellow fever model (1).

Variable	Biological meaning
*S*_*H*_	Density of susceptible humans
*V*_*H*_	Density of vaccinated humans
*I*_*H*_	Density of infected humans
*R*_*H*_	Density of recovered humans
*N*_*H*_	Density of total human population
*S*_*M*_	Density of uninfected mosquitoes
*L*_*M*_	Density of latent mosquitoes
*I*_*M*_	Density of infected mosquitoes
*N*_*M*_	Density of total mosquitoes population
*S*_*E*_	Density of uninfected aquatic forms^*^
*N*_*E*_	Density of total aquatic forms^*^

^*^Those variables are called “aquatic” because mosquitoes spend the large period of their development history in the water [25].

**Table 2:** Description of the parameters of the yellow fever model (1).

Parameter	Biological meaning	Value (baseline)
*a*	Average daily biting rate	3.0

*b*	Fraction of actually infective bites^(+)^	0.6

*μ*_*H*_	Humans natural mortality rate	3.5 × 10^−5^ days^−1^

*r*_*H*_	Birth rate of humans	9.5 × 10^−5^ days^−1^

*k*_*H*_	Humans carrying capacity	5 × 10^6^

*α*_*H*_	Yellow fever mortality in humans	3.5 × 10^−4^ days^−1^

*γ*_*H*_	Humans recovery rate	0.143 days^−1^

*p*	Susceptible eggs hatching rate	0.15 days^−1^

*ω*_*H*_	Rate of waning of immunity induced by vaccination	0.1 days^−1^

υ_*H*_	Vaccination rate	0.5

*f*_*H*_	Vaccine efficacy	0.9

*γ*_*M*_	Mosquitoes latency rate	0.143 days^−1^

*μ*_*M*_	Mosquitoes natural mortality rate	0.09 days^−1^

*r*_*M*_	Oviposition rate	50 days^−1^

*k*_*E*_	Aquatic carrying capacity	9.8 × 10^7^

*μ*_*E*_	Aquatic natural mortality rate	0.1 days^−1^

*c*	*A. aegypti* susceptibility to yellow fever^(&)^	0.8

*c*_*S*_	Climatic factor^(#)^	0.07

^(+)^Probability that an infective bite generates a new infection in humans.

^(&)^Probability that a new infection in the mosquito is generated when it bites an infective host.

^(#)^ *Ad hoc* parameter that modulates seasonality.

**Table 3:** Stability of the equilibrium points of the model (1) for *r*_*H*_ > μ_*H*_.

Conditions	Equilibrium point
μ_*M*_ > μ_*M*_^thres^	*P*_0_^*HM*^ = (*S*_*H*_^0^, *V*_*H*_^0^, 0, 0, 0, 0, 0,0) stable

μ_*M*_ < μ_*M*_^thres^, *r*_*H*_ < *r*_*H*_^thres^	*P*_0_^*HM*^ = (*S*_*H*_^0^, *V*_*H*_^0^, 0,0, 0,0, *N*_M_^*^, *S*_E_^*^) stable

μ_*M*_ < μ_*M*_^thres^, *r*_*H*_ > *r*_*H*_^thres^, υ_*H*_ > υ_*H*_^thres^	*P*_0_^*HM*^ = (*S*_*H*_^0^, *V*_*H*_^0^, 0,0, 0,0, *N*_M_^*^, *S*_E_^*^) stable

μ_*M*_ < μ_*M*_^thres^, *r*_*H*_ > *r*_*H*_^thres^, υ_*H*_ < υ_*H*_^thres^	*P*_1_^*HM*^ = (*V*_H_^*^, *I*_H_^*^, *R*_H_^*^, *L*_M_^*^, *I*_M_^*^, *N*_M_^*^, *S*_E_^*^) stable
